# Mechanistic characterization of waterborne selenite uptake in the water flea, *Daphnia magna*, indicates water chemistry affects toxicity in coal mine-impacted waters

**DOI:** 10.1093/conphys/coad108

**Published:** 2024-01-27

**Authors:** Chantelle E Klaczek, Greg G Goss, Chris N Glover

**Affiliations:** Department of Biological Sciences, CW 405 Biological Sciences Bldg., University of Alberta, Edmonton, Alberta T6G 2E9, Canada; Department of Biological Sciences, CW 405 Biological Sciences Bldg., University of Alberta, Edmonton, Alberta T6G 2E9, Canada; Department of Biological Sciences, CW 405 Biological Sciences Bldg., University of Alberta, Edmonton, Alberta T6G 2E9, Canada; Faculty of Science and Technology and Athabasca River Basin Research Institute, Athabasca University, 1 University Dr., Athabasca, Alberta T9S 3A3, Canada

**Keywords:** Invertebrate, phosphate, transporter, uptake mechanism, waterborne selenite

## Abstract

Concentrations of selenium that exceed regulatory guidelines have been associated with coal mining activities and have been linked to detrimental effects on aquatic ecosystems and the organisms therein. Although the major route of selenium uptake in macroinvertebrates is via the diet, the uptake of waterborne selenite (HSeO_3_^−^), the prominent form at circumneutral pH, can be an important contributor to selenium body burden and thus selenium toxicity. In the current study, radiolabelled selenite (Se^75^) was used to characterize the mechanism of selenite uptake in the water flea, *Daphnia magna*. The concentration dependence (1–32 μM) of selenite uptake was determined in 1-hour uptake assays in artificial waters that independently varied in bicarbonate, chloride, sulphate, phosphate and selenate concentrations. At concentrations representative of those found in highly contaminated waters, selenite uptake was phosphate-dependent and inhibited by foscarnet, a phosphate transport inhibitor. At higher concentrations, selenite uptake was dependent on waterborne bicarbonate concentration and inhibited by the bicarbonate transporter inhibitor DIDS (4,4′-diisothiocyano-2,2′-stilbenedisulfonic acid). These findings suggest that concentrations of phosphate in coal mining-affected waters could alter selenite uptake in aquatic organisms and could ultimately affect the toxic impacts of selenium in such waters.

## Teaser

Coal mining activity is one of the major sources of elevated selenium concentration in aquatic systems; however, little is known about its uptake mechanisms in aquatic organisms. Here, we demonstrate that high waterborne selenite uptake in the water flea, *Daphnia magna*, is likely mediated by a phosphate transporter.

## Introduction

One of the key environmental concerns associated with coal mining is its contribution to elevated water selenium and the resulting toxic effects of selenium on aquatic biota (Lemly, 2004; Etteieb et al., 2020). Indeed, tissue pathologies and teratogenesis have been observed in several field studies of fish species inhabiting waters impacted by coal mining effluents worldwide (e.g. Lemly, 1993, 2014, 2018; Holm et al., 2005; Jasonsmith et al., 2008). For fish, exposure to selenium through the diet is considered to be the route of greatest toxicological significance (e.g. Besser et al., 1993), but studies have shown that the invertebrate prey items that constitute fish diets may exhibit toxicity at selenium body burdens lower than those considered to protect consumer organisms at higher trophic levels (deBruyn and Chapman, 2007). Sublethal effects in freshwater invertebrates include the generation of oxidative stress and the disruption of the important ionoregulatory enzyme sodium/potassium ATPase, with the magnitude of these responses generally corresponding to selenium body burden (Xie et al., 2016).

In most natural freshwaters, selenium exists mainly as selenite (Se(IV)), although in waters of elevated pH and under oxidizing conditions, selenate (Se(VI)) predominates (Conde and Sanz Alaejos, 1997; Torres et al., 2011). The predominant selenium oxidative state also depends on the contributing source. For example, selenite is more prominent in waters receiving discharges from coal ash or oil refineries, whereas selenate dominates in waters receiving discharges from agriculture and copper mining (USEPA, 2016). In microorganisms, selenite appears to have a higher bioavailability than selenate, and once taken up, selenium is accumulated in an organic form, mostly complexed with amino acids (Vandermeulen and Foda, 1988; Brasher and Ogle, 1993; Franz et al., 2011). This organic selenium is therefore the form considered to be of greatest relevance to subsequent trophic transfer. However, it is known that freshwater invertebrates can absorb inorganic selenium directly from the water. For example, significant bioconcentration has been shown in studies exposing larvae of the midge *Chironomus dilutus* to waterborne selenite (Franz et al., 2011; Gallego-Gallegos et al., 2013). In the aquatic oligochaete *Lumbriculus variegatus*, a 14-day waterborne exposure to 15 μg l^−1^ selenite led to a significantly higher body burden than exposure to the same concentration of selenate (Xie et al., 2016). However, this was more than an order of magnitude less than the burdens observed for sediment exposure to 20 μg g^−1^ selenite, representing a dietborne route of uptake (Xie et al., 2016). Indeed, it is well recognized that the diet is the dominant route of selenium uptake, accounting for up to 95% of total selenium accumulation (e.g. Fowler and Benayoun, 1976; Roditi and Fisher, 1999; Schlekat et al., 2004; Lee et al., 2006; Presser and Luoma, 2010), with the relative importance of the waterborne pathway being species-specific, driven by organismal physiology (Presser and Luoma, 2010). Nevertheless, evidence to date therefore indicates that waterborne selenium may contribute to selenium burden, which in turn has toxicological implications for the invertebrate itself and for the organisms that consume it. Consequently, knowledge of the pathways of waterborne selenite uptake is important for characterizing bioavailability and interpreting how changes in water chemistry may affect uptake and toxicity.

In most natural waters, selenite is present as an anion (i.e. HSeO_3_^−^). As such, selenite uptake in aquatic biota is likely to proceed via anionic transport pathways. Indeed, anionic pathways of uptake have been observed in other studied systems. For example, in human erythrocytes, selenite uptake is likely achieved by the bicarbonate/chloride anion exchanger (AE1 or Band 3 or SLC4A1; Kaur et al., 2020), while McDermott et al. (2016) suggested selenite uptake was associated with ZIP8 (SLC39A8) and was transported into a variety of mammalian cells along with zinc and bicarbonate as part of an electroneutral transport process. In plants, considerable research links selenite uptake to phosphate transporters (Zhang et al., 2014; Wang et al., 2019; Bai et al., 2022), and evidence also exists for phosphate transporter-mediated selenite uptake in yeast (Lazard et al., 2010) and in phytoplankton (Araie et al., 2011), although in both groups alternative pathways have also been suggested (monocarboxylate transporters in yeast; McDermott et al., 2010; sulphate and nitrate transporters in phytoplankton; Morlon et al., 2006). In the bacterium *Escherichia coli*, selenite uptake is proposed to be achieved by a sulphate transporter (Lindblow-Kull et al., 1985). In one of the few studies to examine selenite transport in an aquatic eukaryote, Misra et al. (2012) showed that selenite uptake in rainbow trout hepatocytes and enterocytes was affected by the presence of sulphite and the AE1 inhibitor 4,4′-diisothiocyano-2,2′-stilbenedisulfonic acid (DIDS). Consequently, a wide range of putative selenite uptake pathways have been described, varying across organisms and tissues.

In the current study, the mechanism of waterborne selenite uptake was characterized in the water flea, *D. magna*. *Daphnia* perform a critical role in freshwater food chains, being both a primary consumer and a prey item for fish (Miner et al., 2012), and they are a valuable model for studying trace metal handling in aquatic biota (Tsui and Wang, 2007). The sensitivity of *D. magna* to waterborne selenium has been previously characterized, and selenite has been shown to be more toxic than selenate, with 48-h median lethal concentrations of 0.55 and 2.84 mg l^−1^, respectively (Maier et al., 1993). These data reflect the higher bioavailability of waterborne selenite relative to waterborne selenate in this species (Besser et al., 1993). To date, however, there is little mechanistic understanding of how waterborne selenite is taken up across daphnid epithelia. The exception is a single study that showed selenite uptake could be affected by pH and waterborne calcium but remained unimpacted by the presence of waterborne sulphate (Yu and Wang, 2002). To test the hypothesis that, by analogy with other biota, selenite uptake in *D. magna* is achieved via an anion transporter, we employed radiolabelled Se^75^ and a variety of water chemistries to examine short-term selenite uptake. Specifically, using artificial water as a basal medium, all permeant anions were replaced with gluconate salts, and thereafter, concentrations of bicarbonate, chloride, sulphate and phosphate were altered to examine their impacts on selenite uptake. Where effects were shown, inhibitors of specific transporters were used to further probe the mechanism of uptake. The effect of waterborne selenate on selenite uptake was also examined to assess the possibility that the two selenium oxidation states were absorbed through similar mechanisms. Uptake was examined over a range of selenite concentrations (1–32 μM = 79–2527 μg l^−1^) to determine its concentration-dependence. Ultimately, knowledge of the mechanism of selenite uptake in daphnids will facilitate an understanding of how water chemistry may affect the uptake and toxicity of this important aquatic toxicant and may provide insight into conservation approaches for aquatic environments impacted by selenium-rich effluents, such as those associated with coal mining activity.

## Materials and Methods

### Daphnia maintenance

Adult *D. magna* (between 20 and 23 days old; mean (± standard deviation) wet mass = 3.30 ± 0.56 mg) were used for all experiments. These animals were sourced from an established laboratory culture maintained in the Department of Biological Sciences at the University of Alberta. This culture was subjected to a 16 h:8 h light/dark photoperiod at 22°C and cultured in 1-l glass beakers following Organization for Economic Cooperation and Development (OECD) guidelines (OECD, 2004) under the following water chemistry: 2 mM CaCl_2_·2H_2_O; 0.5 mM MgSO_4_·7H_2_O; 0.77 mM NaHCO_3_; 0.08 mM KCl; pH ~7.5. Animals were fed once daily with YCT (a yeast, cereal leaf, trout chow mix) and algae (*Raphidocelis subcapitata*).

### Selenium uptake experiments

The uptake of selenite was examined in a variety of water chemistries (detailed below). Except in the case of testing the effects of inhibitors (see below), in each water chemistry uptake was determined as a function of selenite concentration (1, 2, 4, 8, 16, 32 μM; as sodium selenite from a 1 g l^−1^ stock) over a 1-h exposure period. This concentration range and exposure duration were chosen following preliminary studies that showed uptake was in the linear phase at these concentrations and at this exposure duration. Exposures were conducted in 2-ml microcentrifuge tubes, each containing an individual daphnid (*n* = 8) and 1.5 ml of test water. Uptake was determined using radiolabelled selenite (Se^75^; University of Missouri Research Reactor Centre; 0.1–0.25 μCi per test chamber, varying with ‘cold’ Se concentration). After 1 h of exposure, daphnids were carefully removed from the exposure chambers using a plastic pipette and individually rinsed through a series of three solutions to remove adsorbed, but not absorbed, isotope and to exchange radiolabelled water trapped in the daphnid carapace (2 × 10 s in unlabelled water equivalent to the test water chemistry albeit without selenium, followed by 10 s in 1 g l^−1^ selenite (as sodium selenite)). After rinsing, daphnids were gently blotted dry with tissue paper and weighed using a microbalance (Orion Cahn C-35; Thermo Electron Corporation),

The following water chemistries were examined: 1. OECD culture water (control treatment; composition as above); 2. ‘Gluconate’ (water with no permeant anions; 0.77 mM C_6_H_11_NaO_7_ and 0.08 mM C_6_H_11_KO_7_ as the only salts); 3. ‘Bicarbonate’ (OECD water without NaHCO_3_); 4. ‘Chloride’ (two chemistries, one with chloride salts removed from OECD water and one where chloride salts were doubled in concentration relative to OECD water); 5. ‘Sulphate’ (OECD water without MgSO_4_·7H_2_O); 6. ‘Phosphate’ (OECD water supplemented with 10, 100 or 1000 μM Na_3_PO_4_). All test chemistries used ultrapure (>18 MΩ) water as a base, and all had pH adjusted to pH 7.5 using potassium hydroxide, hydrochloric acid (all treatments except ‘chloride’) and/or sulphuric acid (‘chloride’ water chemistries). No attempts were made to correct for ionic strength or to hold cation concentrations identical across all treatments. Preliminary studies indicated that there were minor fluctuations of selenite uptake rates over time, and thus all test water chemistries were run concurrently with a time-matched OECD water control. Selenium speciation in all experimental waters was modelled using Visual MINTEQ.

After weighing, daphnids were then individually placed in 12 × 75-mm borosilicate culture tubes, and radioactivity (i.e. counts per minute) was measured using a Cobra Quantum gamma counter. All radioactivity counts were transformed to pmol of selenite by dividing counts per minute (cpm) by water specific activity (cpm pmol^−1^). This value was then divided by daphnid wet mass to give pmol mg wet weight^−1^.

### Inhibitor experiments

These studies were all conducted using OECD water as the base medium, to which selenite was added from 1 g l^−1^ selenite (as sodium selenite) solution. All studies were 1 h in length and proceeded as described above, unless specifically noted otherwise below.

The effects of DIDS (≥85%, Sigma-Aldrich, St. Louis, MO), a bicarbonate transport inhibitor, was tested at selenite concentrations of 1, 8 and 32 μM, chosen based on the outcomes of the ‘bicarbonate’ test series. Stock solutions of DIDS at 1000× working concentrations suspended in dimethyl sulfoxide (DMSO) were made immediately prior to exposures and added to test solutions immediately prior to daphnid addition for final concentrations of 200, 500 and 1000 μM. This highest test concentration has previously been applied in adult *D. magna* (Bianchini and Wood, 2008). The time-matched controls were inoculated with an equivalent concentration (0.001%) of DMSO.

Nicotinamide adenine dinucleotide (NAD^+^; ≥95%, Sigma-Aldrich, St. Louis, MO) was added to 2 μM selenite at a concentration of 300 μM, immediately prior to daphnid addition. NAD^+^ is a putative phosphate transporter inhibitor, and 300 μM has been shown to effectively inhibit phosphate transport in rat membrane vesicle studies (Kempson et al., 1981). Sodium phosphonoformate (PFA, aka Foscarnet; Sigma-Aldrich St. Louis, MO), is another putative phosphate transporter inhibitor, and was added to 2 μM selenite at concentrations of 1, 10 or 50 mM immediately prior to daphnid addition. These concentrations are in the range where effects on phosphate transport have been previously observed in rainbow trout and hagfish (Avila et al., 2000; Schultz et al., 2014).

To test whether selenate and selenite were using the same transporter for uptake in *D. magna*, the effects of waterborne selenate on selenite uptake were examined. To maximize the capacity for selenate inhibition, effects of 4 and 32 μM selenate (from a 1 g l^−1^ sodium selenate stock) were examined in the presence of 0.75 nM selenite (representing only selenite added with 0.1 μCi of the radiolabelled stock and no ‘cold’ selenite addition). Because of the low selenite concentration examined, the final units for selenite uptake in this study were expressed in pmol g wet weight^−1^.

### Statistics

Significant differences between treatments and time-matched controls were assessed within each tested selenite concentration using a one-way ANOVA, with a Holm-Sidak *post hoc* test. Since this resulted in multiple analyses within each dataset, a Bonferroni multiple comparisons correction was applied, leading to different alpha levels for each analysis. A two-way ANOVA, where effects of treatment and concentration were simultaneously assessed, could not be used owing to lack of normality and inequality of variance, as determined by Shapiro–Wilk and Brown-Forsythe tests, respectively. Data transformations failed to render these data appropriate for parametric analysis. Shapiro–Wilk and Brown-Forsythe analyses were also used to interrogate the data prior to conducting one-way ANOVAs, and where data failed either test, a transformation was performed until the assumptions of normality and equality of variance were met, or a non-parametric Kruskal–Wallis ANOVA was conducted. The specific test and/or transformation used and the alpha value for each dataset in the manuscript are reported in Supplemental Table 1. All statistical analyses were performed using SigmaPlot (ver. 14.5; Systat Software Inc.).

## Results

Varying the exposure water chemistry did not affect selenite speciation at pH 7.5 as determined by Visual MINTEQ. Exposures with selenite (Se(IV)) resulted in HSeO_3_^−^ (84%) and SeO_3_^2−^ (16%) being the dominant species. In the study with added selenate (Se(VI)), SeO_4_^2−^ (96%) and CaSeO_4_ (4%) were the dominant species. Selenium speciation did not vary significantly as a function of exposure concentration over the range of concentrations tested.

**Figure 1:**
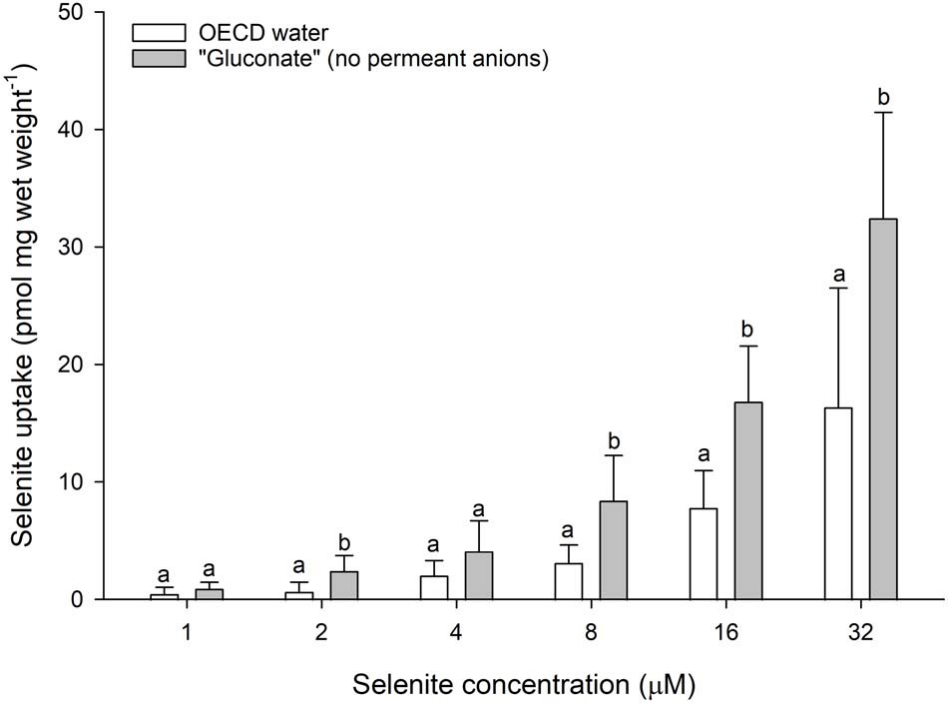
Selenite uptake (pmol mg wet weight^−1^) in daphnids as a function of waterborne selenite concentration following a 1-h exposure to water without any permeant anions (‘Gluconate’, 0.77 mM C_6_H_11_NaO_7_ and 0.08 mM C_6_H_11_KO_7_; grey bars) or OECD water (control, white bars). Plotted points represent the means (± standard deviation) of eight replicates. Bars sharing letters within each selenite concentration are not statistically significantly different, as determined by a one-way ANOVA followed by a *post hoc* Holm–Sidak test.

To confirm whether selenite uptake was anion-dependent, we exposed daphnids to a water chemistry where all permeant anions were replaced by gluconate. These data showed elevated selenite uptake rates in the gluconate water chemistry relative to the OECD water at selenite concentrations of 2, 8, 16 and 32 μM (Fig. 1; see Supplemental Table 1 for statistical outcomes).

**Figure 2:**
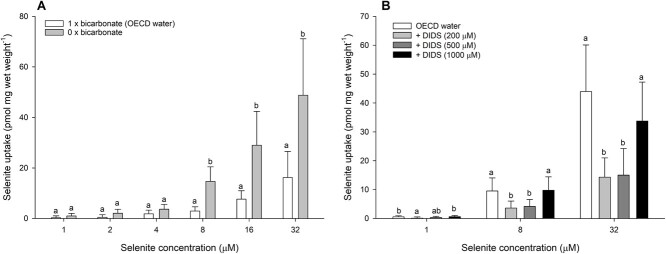
Selenite uptake (pmol mg wet weight^−1^) in daphnids as a function of waterborne selenite concentration following a 1-h exposure to OECD water without (0 mM, grey bars) or with (0.77 mM, white bars) sodium bicarbonate (A), and in OECD water with varying DIDS concentration (0 mM, white bars; 200 μM, light grey bars; 500 μM, dark grey bars; 1000 μM, black bars; B). Plotted points represent the means (± standard deviation) of 8 replicates. Bars sharing letters within each selenite concentration are not statistically significantly different, as determined by a one-way ANOVA followed by a *post hoc* Holm–Sidak test or Kruskal–Wallis ANOVA (32 μM Se, Panel B).

To identify which anions may have been responsible for limiting selenite uptake in the gluconate experiment, a series of tests were conducted where the anion concentrations in the test water chemistry were varied. In the absence of sodium bicarbonate, there was no significant effect on selenite uptake relative to the OECD water control containing 0.77 mM NaHCO_3_ at low selenite concentrations (1, 2 and 4 μM). However, at high selenite concentrations (8, 16 and 32 μM), the presence of bicarbonate significantly reduced selenite uptake to values between 32% and 50% of the bicarbonate-free treatment (Fig. 2A; Supplemental Table 1). The addition of 200 μM and 500 μM DIDS significantly reduced selenite uptake at 8 μM and 32 μM, while only the former significantly reduced selenite uptake at 1 μM (Fig. 2B; Supplemental Table 1).

**Figure 3:**
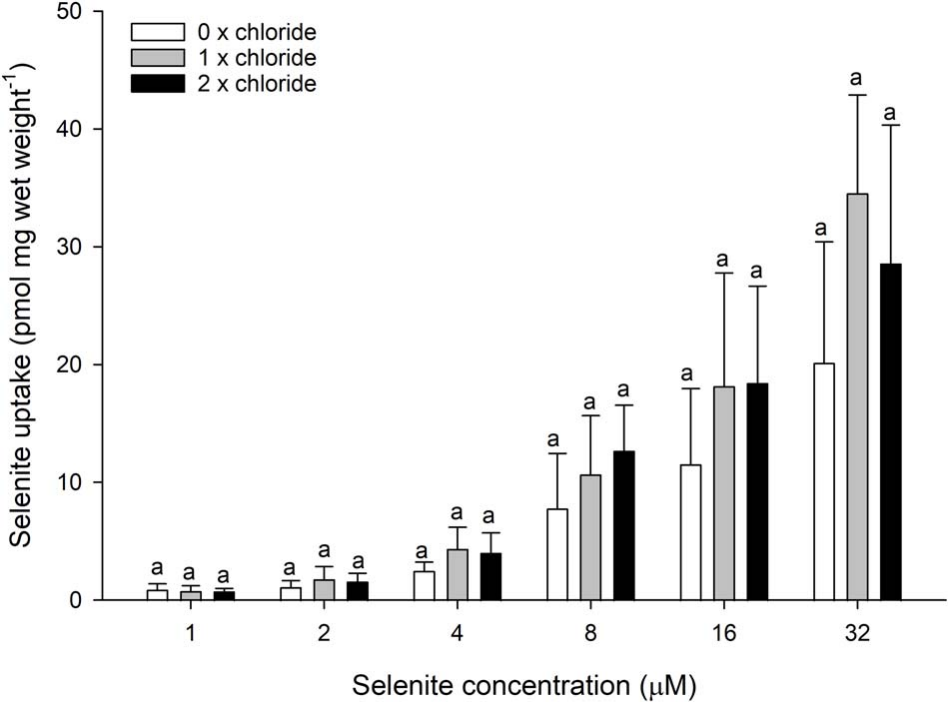
Selenite uptake (pmol mg wet weight^−1^) in daphnids as a function of waterborne selenite concentration following a 1-h exposure to OECD water with varying chloride concentration (0 mM, white bars; 2 mM CaCl_2_·2H_2_O + 0.08 mM KCl, grey bars; 4 mM CaCl_2_·2H_2_O + 0.16 mM KCl, black bars). Plotted points represent the means (± standard deviation) of 8 replicates. Bars sharing letters within each selenite concentration are not statistically significantly different, as determined by a one-way ANOVA followed by a *post hoc* Holm–Sidak test.

**Figure 4:**
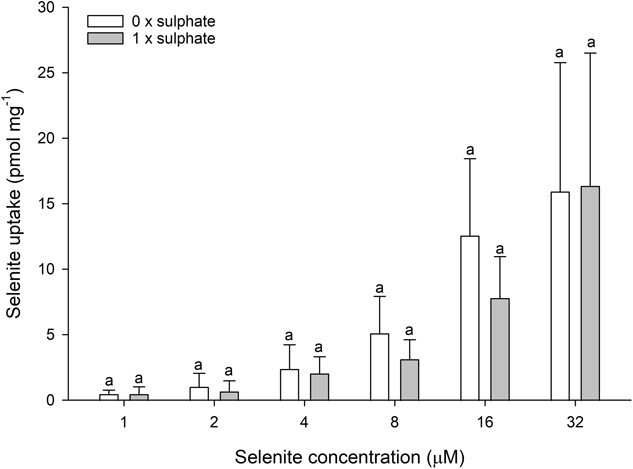
Selenite uptake (pmol mg wet weight^−1^) in daphnids as a function of waterborne selenite concentration following a 1-h exposure to OECD water with varying sulphate concentration (0 mM, white bars; 0.5 mM MgSO_4_·7H_2_O, grey bars). Plotted points represent the means (± standard deviation) of 8 replicates. Bars sharing letters within each selenite concentration are not statistically significantly different, as determined by a one-way ANOVA followed by a *post hoc* Holm–Sidak test.

In contrast to the effect of water bicarbonate, altering the chloride concentration of the water had no effect on selenite uptake (Fig. 3). A similar lack of effect was observed for sulphate, where removing magnesium sulphate from the OECD water had no significant impact on waterborne selenite uptake across the tested selenite concentration range (Fig. 4).

**Figure 5:**
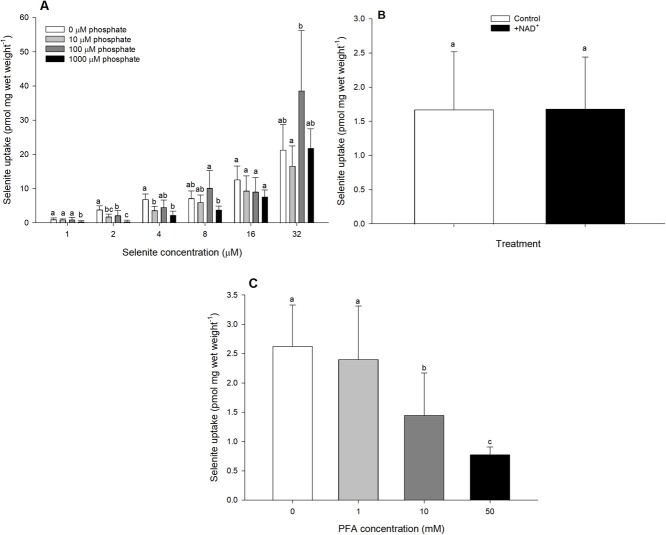
Selenite uptake (pmol mg wet weight^−1^) in daphnids as a function of waterborne selenite concentration following a 1-h exposure to OECD water with varying sodium phosphate concentrations (0 μM, white bars; 10 μM, light grey bars; 100 μM, dark grey bars; 1000 μM, black bars; A), or in the presence of putative phosphate transporter inhibitors NAD^+^ (0 μM, white bar; 300 μM, black bar; B) or PFA (0 mM, white bar; 1 mM, light grey bar; 10 mM, dark grey bar; 50 mM black bar; C). Plotted points represent the means (± standard deviation) of 8 replicates. Bars sharing letters within each selenite concentration are not statistically significantly different, as determined by a one-way ANOVA followed by a *post hoc* Holm–Sidak test.

The addition of phosphate to OECD water had a significant effect on selenite uptake. At selenite concentrations of 4 μM or lower, 1000 μM phosphate significantly reduced selenite uptake relative to the phosphate-free control (Fig. 5A; Supplemental Table 1). Indeed, at 1 µM selenite uptake was reduced by up to 36% of the control value. Additionally, at 2 and 4 μM, 10 μM phosphate significantly reduced selenite uptake (Fig. 5A; Supplemental Table 1). The addition of 300 μM NAD^+^ failed to affect selenite uptake when tested at a waterborne selenite concentration of 2 μM (Fig. 5B), but 10 and 50 mM PFA/foscarnet significantly reduced selenite uptake at this selenite concentration (Fig. 5C; Supplemental Table 1). This effect was dose-dependent, with 10 mM PFA reducing selenite uptake by 45%, and 50 mM PFA reducing selenite uptake by 70%.

**Figure 6:**
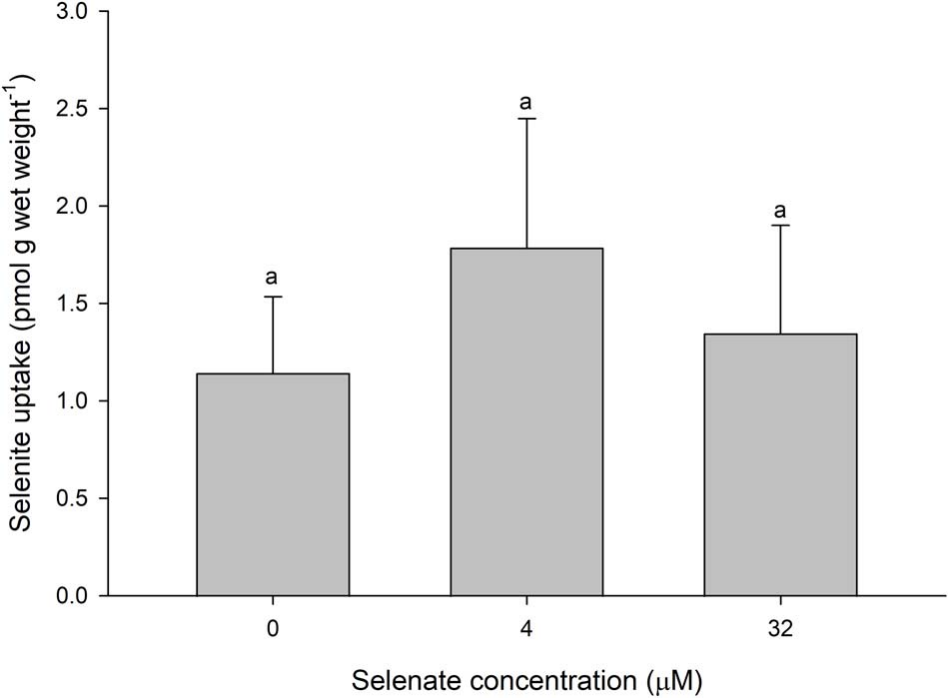
Selenite uptake (pmol g wet weight^−1^) in daphnids as a function of waterborne selenate concentration following a 1-h exposure to OECD water. Plotted points represent the means (± standard deviation) of 8 replicates. Bars sharing letters within each selenite concentration are not statistically significantly different, as determined by a one-way ANOVA followed by a *post hoc* Holm–Sidak test.

The addition of waterborne selenate had no effect on selenite uptake (Fig. 6; Supplemental Table 1). This effect persisted for both tested waterborne selenate concentrations (4 and 32 μM) and despite a large molar excess of selenate relative to the very low selenite concentration in the water (0.75 nM).

## Discussion

The uptake of waterborne selenite in *D. magna* is anion-dependent. As evidence for this, the removal of all permeant anions from the OECD culture water (i.e. no bicarbonate, chloride or sulphate) in the gluconate treatment resulted in increased selenite uptake compared to uptake in the OECD medium containing permeant anions. This suggests that at least one of the anions in OECD water likely competes with selenite for uptake. This hypothesis, and the putative identity of competing anions, is discussed further below.

It is important to note that gluconate (added as a replacement for permeant anions in this study) is known to bind cations (Christoffersen and Skibsted, 1975). This is an important observation because Yu and Wang (2002) showed that selenite uptake in *D. magna* is calcium-dependent. However, at the gluconate concentrations (<1 mM) and the high ratio of calcium: gluconate (~2.3:1) used in the current study, calcium binding by gluconate would have been negligible (Leonhard-Marek et al., 2007). Furthermore, the chloride experiment provides indirect support for a lack of effect of calcium. In this study, the levels of CaCl_2_·2H_2_O ranged from 0 to 4 mM, and although this experiment was designed to test the effect of the anion, it also shows that there was no effect associated with the variation in calcium. This contrast with the study of Yu and Wang (2002) may be a consequence of a much lower selenite concentration over a much longer exposure period in the previous work than in the current study (0.016–0.643 μM vs 1–32 μM and 12 h vs 1 h), reflecting indirect responses of calcium (e.g. on membrane permeability; Yu and Wang, 2002), rather than effects directly related to interactions at a selenite transporter. Overall, therefore, it is more likely that the effects of gluconate on selenite uptake reflect the impact of permeant anion removal, rather than changes in cation bioavailability.

The inhibition of selenite uptake at low concentrations (≤4 μM; 316 μg l^−1^) by phosphate addition to the test water indicated that this anion was a key competitor. This hypothesis that selenite uptake likely occurs through a phosphate transporter is consistent with studies on selenite uptake in plants and yeast (Lazard et al., 2010; Araie et al., 2011; Zhang et al., 2014; Vriens et al., 2016; Wang et al., 2019; Bai et al., 2022). The finding of selenite uptake in *D. magna* via a phosphate transporter is further supported by the effect of application of the phosphate transporter inhibitor PFA/foscarnet, wherein increased levels of this blocker reduced selenite uptake. However, these results were not supported by the outcomes of an experiment with another putative phosphate transporter blocker, NAD^+^, which failed to inhibit selenite uptake. These inhibitors both target sodium–phosphate transporters such as those of the SLC34 family; however, they have distinct putative modes of action. It is suggested that PFA has a competitive effect directly blocking the uptake site, whereas NAD^+^ acts non-competitively by indirectly effecting transporter function through transporter modification (Loghman-Adham, 1996; Sorribas et al., 2019; Lucea et al., 2022). While evidence exists for sodium-dependent phosphate transporters in daphnids (e.g. National Center for Biotechnology Information Accession No. NC_059183.1), the relative efficacy of different inhibitors of the daphnid sodium-dependent phosphate transporters remains uncharacterized.

Our study provides evidence for selenite uptake through a phosphate transporter at selenite concentrations <4 μM (316 μg l^−1^), with our lowest test concentration of 1 μM (79 μg l^−1^). Although selenate is the predominant oxidation state of selenium in most effluent-impacted waters (Martin et al., 2011), waterborne selenite concentrations approaching our lowest test concentration have been reported. For example, a selenite concentration of 57 μg l^−1^ (Etteieb et al., 2021) has been measured associated with mine effluents in Quebec, Canada. For context, water quality guidelines for total selenium in North America range between 1 and 3.1 μg l^−1^ depending on jurisdiction and water body type (see Environment and Climate Change Canada, 2022). Consequently, the selenite concentrations tested in the current study are of the same order of magnitude as elevated environmental concentrations. It is also worth highlighting that the phosphate concentrations used in the current study are also elevated relative to most natural waters. For example, our lowest test concentration of 10 μM would be considered at the higher end of the range of naturally occurring phosphate concentrations in North American lakes (0.03–17 μM; Hudson et al., 2000). However, the phosphate concentrations were selected relative to the experimental selenite concentrations, which, as noted above, are high. Therefore, the key measure here is the ratio of phosphate to selenite. In the current work, phosphate inhibition of selenite uptake in *D. magna* occurred at ratios as low as 2.5:1 (10 μM phosphate vs. 4 μM selenite). Selenite concentrations in contaminated waters are usually in the order of 0.15 μM (Bujdoš et al., 2005; Lashari et al., 2022). Thus, phosphate concentrations in the vicinity of 0.5 μM are likely to have an effect on daphnid selenite uptake, assuming that the use of the putative phosphate transporter is conserved at lower selenite concentrations. Phosphate could be even more effective at inhibiting selenite uptake, as lower concentrations of phosphate were not tested. For example, in the yeast *Saccharomyces cerevisiae*, phosphate and selenite were shown to share uptake pathways, but the two transporters characterized were 35 to 1260 times more specific for phosphate than for selenite (Lazard et al., 2010). A full kinetic characterization of phosphate uptake in the presence and absence of selenite (and of selenite uptake in the absence and presence of phosphate) would be required to draw conclusions regarding the relative affinities of the daphnid transporters for these two substrates. In general, therefore, lower, more environmentally relevant selenite and phosphate concentrations were not tested in the current study, and the analysis of selenite uptake at such concentrations would be required to further confirm the broader environmental and conservation value of this finding.

Altering water bicarbonate concentration also impacted waterborne selenite uptake in *D. magna.* Our results demonstrated that at relatively high selenite concentrations (8–32 μM; 632–2527 μg l^−1^) bicarbonate can partially block selenite uptake. Bicarbonate dependence of selenite uptake has been suggested in human erythrocytes, where selenite demonstrated affinity for the AE1 (Band 3) bicarbonate/chloride transporter (Galanter et al., 1993; Kaur et al., 2020). Evidence from the current study supports this, with the addition of DIDS, an inhibitor of AE1 (Romero et al., 2004), significantly reducing selenite uptake. In our study, the effects of DIDS were only seen at the two lower test concentrations (200 and 500 μM) and not at the highest test concentration (1 mM). Previous work has also shown 1 mM DIDS to be ineffective in modifying daphnid sodium transport (Bianchini and Wood, 2008), but lower concentrations were not tested. We speculate that the lack of dose dependency of DIDS may relate to non-specific effects on other transport pathways in daphnids, as observed in other species at high DIDS concentrations (Cabantchik and Greger, 1992). Importantly, there is also support for DIDS inhibition of selenite accumulation in rainbow trout (Misra et al., 2012), suggesting that this may be a conserved pathway for selenite uptake. However, at least for daphnids, this pathway is unlikely to be of any environmental relevance, given its presence only at very high selenite concentrations (>632 μg l^−1^).

The lack of effect of water chloride manipulation argues against selenite uptake being achieved through a bicarbonate/chloride transporter. However, there are several key points of evidence to consider. First, DIDS does not completely inhibit selenite uptake (application of 200 and 500 μM DIDS reduces selenite uptake to between 33% and 67% of the uninhibited control), indicating that a component of selenite transport occurs independently of DIDS-sensitive transporters. This could even be indicative of the previously noted phosphate transporter that may become more important at high selenite concentrations in the absence of a viable bicarbonate-related pathway. Second, the addition of DIDS is likely to be a more effective intervention than removing water chloride. Removing chloride from the water does not remove chloride from the animal. Therefore, the presence of chloride in daphnid tissues may be sufficient to maintain the actions of a bicarbonate/chloride exchange even in the absence of water chloride. Third, DIDS is known to inhibit other bicarbonate transporters in the SLC4 family, such as sodium/bicarbonate exchangers (Romero et al., 2004). Indeed, evidence in fish suggests that sodium/bicarbonate exchange may be a pathway of selenite uptake in enterocytes and hepatocytes (Misra et al., 2012). Taken together, the effects of DIDS and the lack of effect of water chloride indicate that a bicarbonate exchanger of the SLC4 family may be the mediator of the bicarbonate-dependent selenite uptake in *D. magna*.

Similar to the lack of effect of chloride, changing water sulphate also had no impact on selenite uptake in *D. magna*. This indicates that sulphate transporters are unlikely to be involved in selenite uptake in this species. This is in accordance with multiple other studies that demonstrate selenite is not sulphate-dependent. This is in contrast to the uptake of selenate, which is shown to be sulphate-dependent in a number of different study systems (Brown and Shrift, 1980; Hansen et al., 1993; Ogle and Knight, 1996; Selinus, 2013; Vriens et al., 2016), including *D. magna* (Yu and Wang, 2002).

As selenate uptake occurs via sulphate transporters and selenite uptake is sulphate-independent, it is no surprise that there was a lack of effect of selenate on selenite uptake in our study. The lack of effect also suggests that there is no significant conversion of selenate to selenite, for example by chemical or enzymatic reduction in the vicinity of the epithelial surface. This is a phenomenon that has been shown to occur for the uptake of copper and iron in aquatic biota (Bury et al., 2003).

## Conclusion

The current study demonstrated that *D. magna* takes up waterborne selenium in the form of selenite. Furthermore, at selenite concentrations characteristic of highly contaminated systems, water phosphate addition inhibited selenite uptake. At even higher selenite concentrations, waterborne bicarbonate blocked uptake. The former observation is likely mediated by a sodium–phosphate transporter, as blockade of this transporter by the phosphate transporter inhibitor PFA/foscarnet also blocked selenite uptake. The effect of water bicarbonate was blocked by the AE1 inhibitor DIDS but not affected by water chloride, suggesting that selenite uptake may be mediated by a DIDS-sensitive sodium/bicarbonate transporter. Therefore, further studies are necessary to better clarify the mechanism by which bicarbonate interferes with selenite uptake in daphnids. Further work also needs to examine selenite uptake at more environmentally realistic concentrations to determine if the effects of phosphate persist in natural settings. Critically, however, the evidence to date suggests that the concentration of phosphate in receiving water could provide protection against the accumulation of selenite in zooplankton affected by coal mining effluents. Such an effect would likely offset potential selenium toxicity and limit biomagnification at higher trophic levels, protecting biodiversity in impacted waterways.

## Supplementary Material

Web_Material_coad108

## Data Availability

The data underlying this article are available in the article, in its online supplementary material and will be shared on reasonable request to the corresponding author.
