# Supplementary material for: Mechanistic characterization of waterborne selenite uptake in the water flea, Daphnia magna, indicates water chemistry affects toxicity in coal mine-impacted waters
Source: Conserv Physiol. 2024 Jan 27;12(1):coad108. doi: 10.1093/conphys/coad108 (PMC10823350; doi:10.1093/conphys/coad108)
Supplement: Web_Material_coad108 [file web_material_coad108.zip › Supplemental Material_Daphnia_111423.pdf]

Supplemental Material

Mechanistic characterisation of waterborne selenite uptake in the water flea, *Daphnia magna*, indicates water chemistry impacts toxicity in coal mine-impacted waters

Chantelle E. Klaczek[1,*], Greg G. Goss[1], Chris N. Glover[1,2]

[1]Department of Biological Sciences, University of Alberta, Edmonton, Alberta, Canada T6G 2E9

[2]Faculty of Science and Technology and Athabasca River Basin Research Institute

Supplemental Table 1: Statistical approach and outcomes for selenite uptake data as a function of experimental water chemistry

**Treatment**	**Selenite concentration (μM)**	**Data transformation**	**Bonferroni-adjusted alpha level**	**P value**
Gluconate	1	Log	0.0083	0.046
	2		0.0083	**0.005**
	4		0.0083	0.059
	8		0.0083	**0.004**
	16		0.0083	**<0.0001**
	32		0.0083	**<0.0001**
Bicarbonate	1	Log	0.0083	0.088
	2		0.0083	0.012
	4		0.0083	0.013
	8	Log	0.0083	**<0.0001**
	16	Log	0.0083	**<0.0001**
	32	Log	0.0083	**<0.0001**
DIDS	1		0.017	**0.003**
	8		0.017	**0.001**
	32	KW ANOVA	0.017	**<0.001**
Chloride	1	Log	0.0083	0.773
	2		0.0083	0.223
	4		0.0083	0.044
	8		0.0083	0.117
	16		0.0083	0.187
	32		0.0083	0.032
Sulphate	1	Log	0.0083	0.414
	2	Square Root	0.0083	0.464
	4		0.0083	0.664
	8		0.0083	0.122
	16		0.0083	0.063
	32		0.0083	0.932
Phosphate	1	Log	0.0083	**<0.0001**
	2		0.0083	**<0.0001**
	4		0.0083	**<0.0001**
	8	Log	0.0083	**<0.0001**
	16		0.0083	0.075
	32	Log	0.0083	**<0.0001**
Selenate	0.75 nM		0.05	0.078
NAD^+	2		0.05	0.978
PFA	2	Log	0.05	**<0.001**

Bolded values in p-value column represent statistically significant data.
